# Data-driven multilayer complex networks of sustainable development goals

**DOI:** 10.1016/j.dib.2019.104049

**Published:** 2019-05-23

**Authors:** Viktor Sebestyén, Miklós Bulla, Ákos Rédey, János Abonyi

**Affiliations:** a Institute of Environmental Engineering University of Pannonia, Veszprém, Hungary; b National Environmental Council (NEC), Budapest, Hungary; c MTA-PE “Lendület” Complex Systems Monitoring Research Group, University of Pannonia, Veszprém, Hungary

**Keywords:** Sustainability, Sustainable development goals, Network analysis, Data driven modelling, Evidence-based decision making, Spatial analysis

## Abstract

This data article presents the formulation of multilayer network for modelling the interconnections among the sustainable development goals (SDGs), targets and includes the correlation based linking of the sustainable development indicators with the available long-term datasets of The World Bank, 2018 [1]. The spatial distribution of the time series data allows creating country-specific sustainability assessments. In the related research article “Network Model-Based Analysis of the Goals, Targets and Indicators of Sustainable Development for Strategic Environmental Assessment” [2] the similarities of SDGs for ten regions have been modelled in order to improve the quality of strategic environmental assessments. The datasets of the multilayer networks are available on Mendeley [3].

**Table:** Specifications table

Subject area	*Network theory, sustainable development, sustainability, spatial assessment*
More specific subject area	*Network model, interconnections of sustainable development goals, country-specific sustainability assessment*
Type of data	*Microsoft Excel spreadsheet (XLS), MATLAB code, text file*
How data was acquired	*The linking of the indicators has been done based on the correlation of the available data. The network files are generated from the correlation among the indicators. The top-down structure of the goals, targets and indicators are available in the literature*[4], [5]*.*
Data format	*Generated, analysed*
Experimental factors	*N/A*
Experimental features	*The multilayer networks (goals, targets and indicators) have been created with the correlation of the World Bank's variables.*
Data source location	*263 different geographical unit*
Data accessibility	*The data are available in this article and publicly on Mendeley*[3].
Related research article	*Sebestyén, V., Bulla, M., Rédey, Á., Abonyi, J.: Network Model-Based Analysis of the Goals, Targets and Indicators of Sustainable Development for Strategic Environmental Assessment, Journal of Environmental Management, Vol. 238, pp. 126–135,*https://doi.org/10.1016/j.jenvman.2019.02.096*.*

**Table:** 

**Value of the Data** • The dataset can be used to model the synergies and trade-offs among sustainable development goals, targets and indicators. • The networks of the SDGs are available for every country and geographical regions can support country-specific assessments. • The generated networks of data are suitable for identifying thematic areas and key indicators of long-term sustainability planning. • The network can be used as a benchmark example for researchers interested in the development of analysis of multilayer networks.

## 1 Data

The dataset presented in this paper defines multilayer networks formed based on time series of macroeconomic variables (“Worldbank all data.xlsx”) linked to the indicators and targets of sustainable development goals (SDGs). The 530 links connecting the World Bank variables [1] with SDG indicators are listed in the “edge_indicator_variable.xlsx” excel file. The files representing the networks are generated for the analysis in MuxViz, which is a framework for the multilayer analysis and visualization. The first Matlab program VV_study_v1.m calculates the interconnections among the World Bank variables and creates the network files for the further assessments with the WB_preprocess_v1.m Matlab file. The indirect linking of the SDGs (goals to goals and targets to targets) and formulation of the network files is done in the third tool (preprocess_mat_v2.m). The following network files were generated by the own developed MuxViz exporter tool. formulated for analysis in MuxViz:

• Countries_WB_Config.txt: The structure of the multilayer network that includes the relationships between the variables of a given country.
• SDG_VV_Config.txt: The interconnections of the World Bank variables.
• SDG_TIV_VIT_Config.txt: The interconnections of the SDG targets based on the World Bank Open Data.
• SDG_GTIV_VITG_Config.txt: The interconnections of the SDG goals based on the World Bank Open Data.
• SDG_TT_Config.txt: The interconnections of the SDG targets based on the study of International Council for Science.
• SDG_GT_TG_Config.txt: The interconnections of the SDG goals based on the study of International Council for Science.

**Fig. 1:**
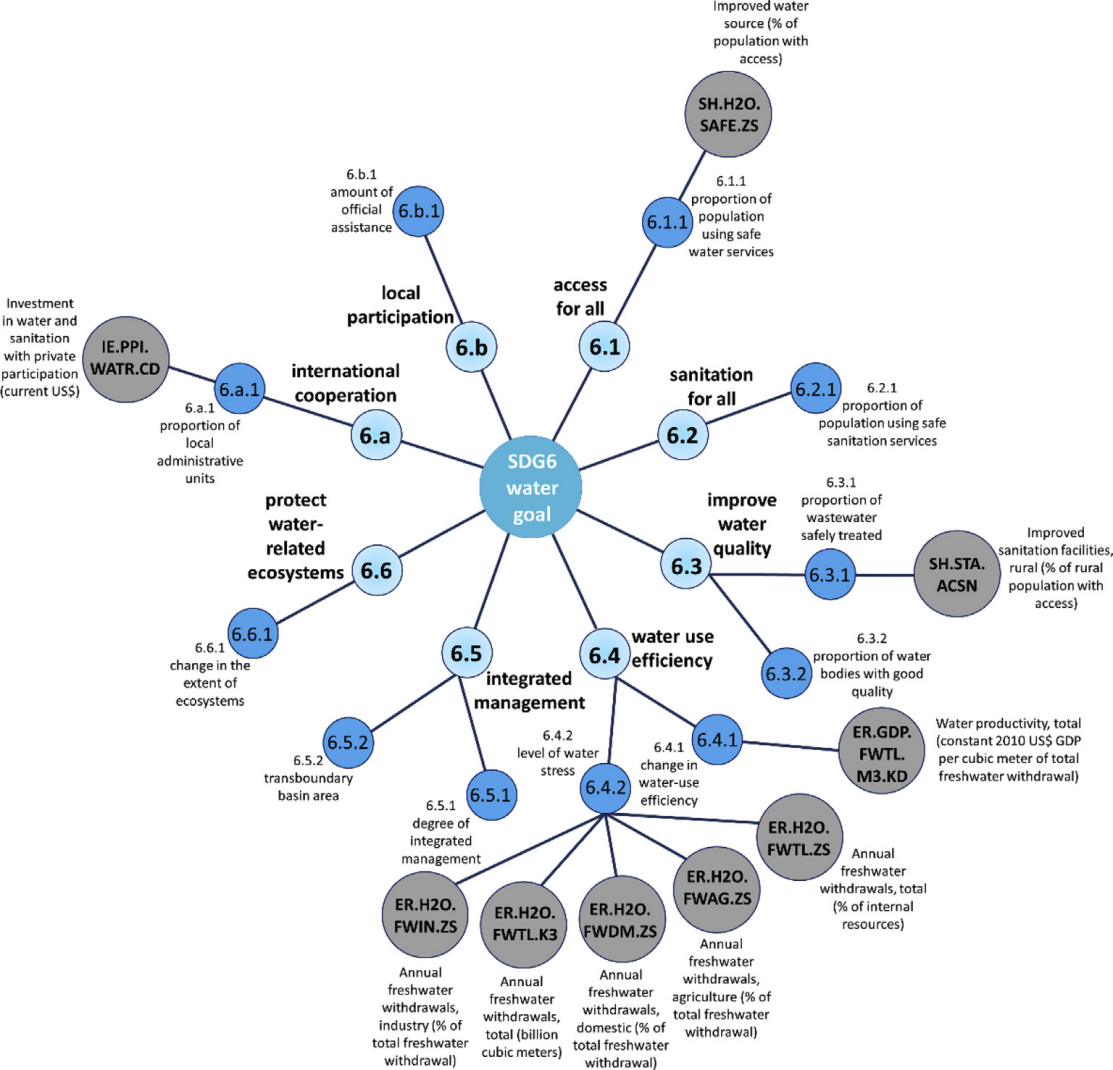
The linking of World Bank variables with the SDG6 “water goal”.

**Fig. 2:**
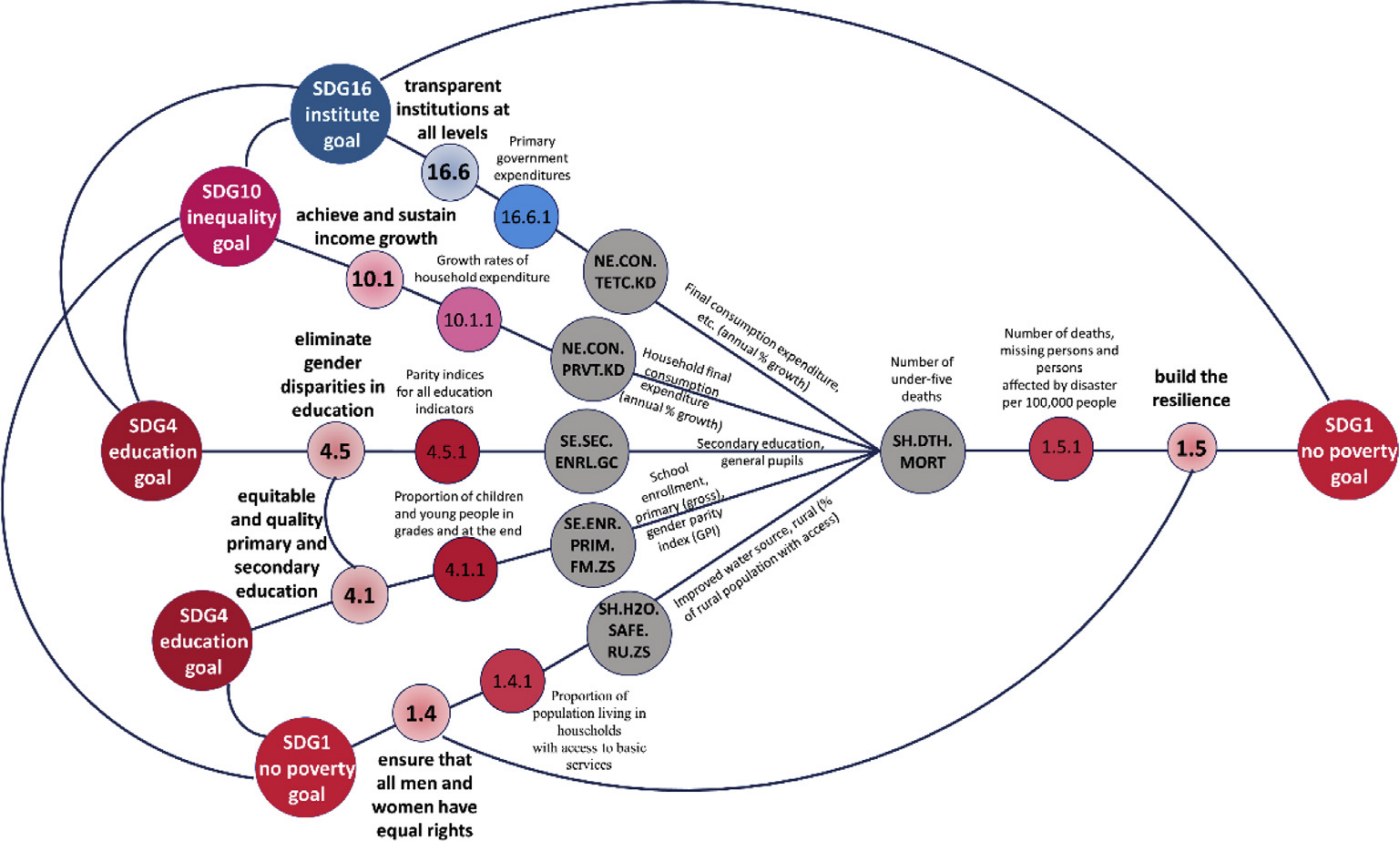
The indirect linking of the sustainable development goals.

**Fig. 3:**
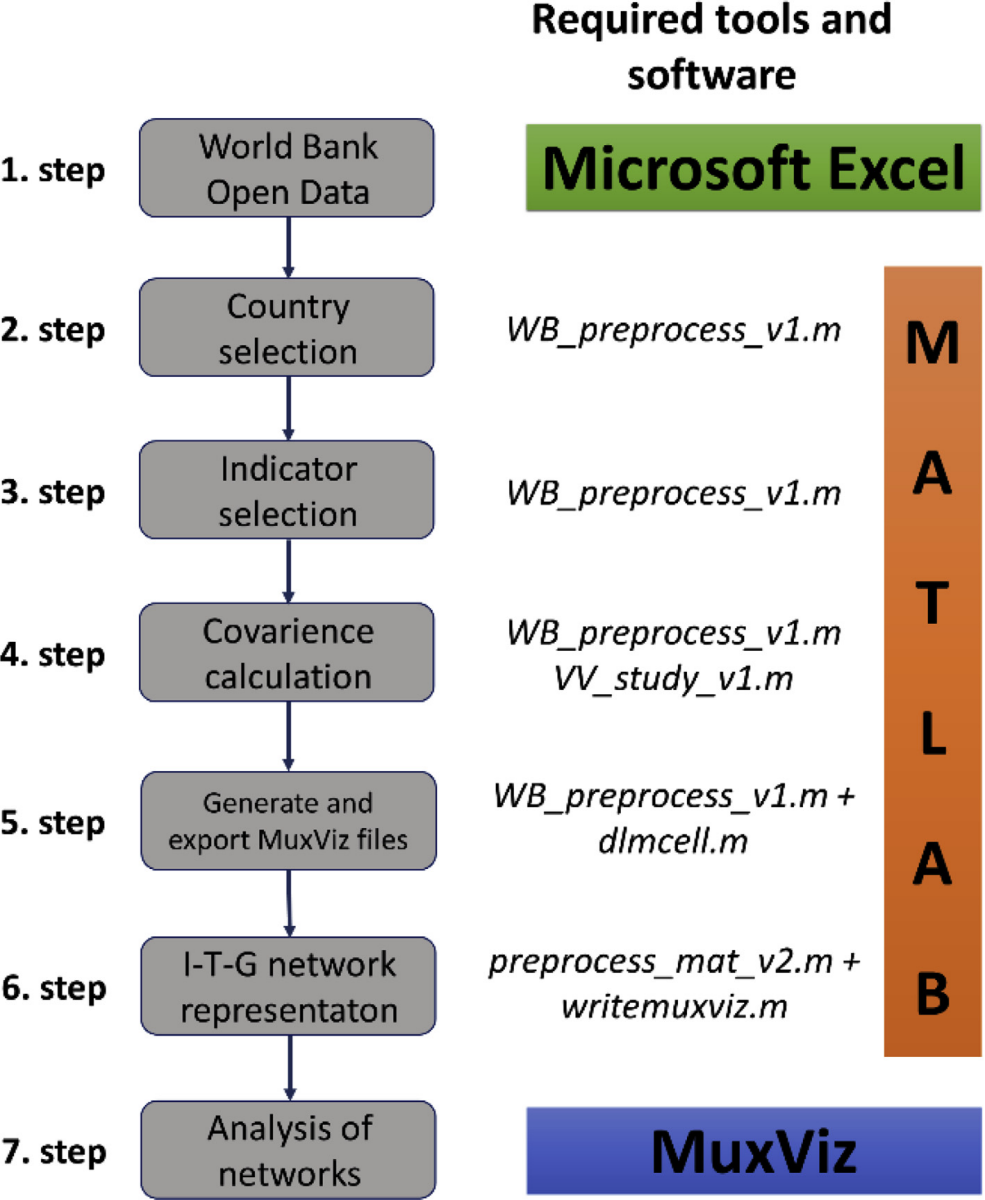
The workflow of the modelling of SDG interconnections.

The structure of the networked data is illustrated in Fig. 1 that shows the how the time series of World Bank Open Data [1] are linked to the sixth sustainable development goal. Based on the correlation calculations and the “top-down” structure of the sustainable development goals, targets and indicators the indirect linking of the targets and goals were completed, as it can be seen on Fig. 2. Fig. 3 summarizes how the Matlab codes generate the networks. In this data article the following regions were selected for the analysis: the World (WLD), members of the Organisation for Economic Co-operation and Development (OECD) (OED), the Arab World (ARB), Central Europe and the Baltics (CEB), East Asia & Pacific (EAS), East Asia & Central Asia (ECS), the European Union (EUU), Latin America & the Caribbean (LCN), the Middle East & North Africa (MEA), and North America (NAC). The MATLAB code refers to the abbreviations in parentheses.

**Fig. 4:**
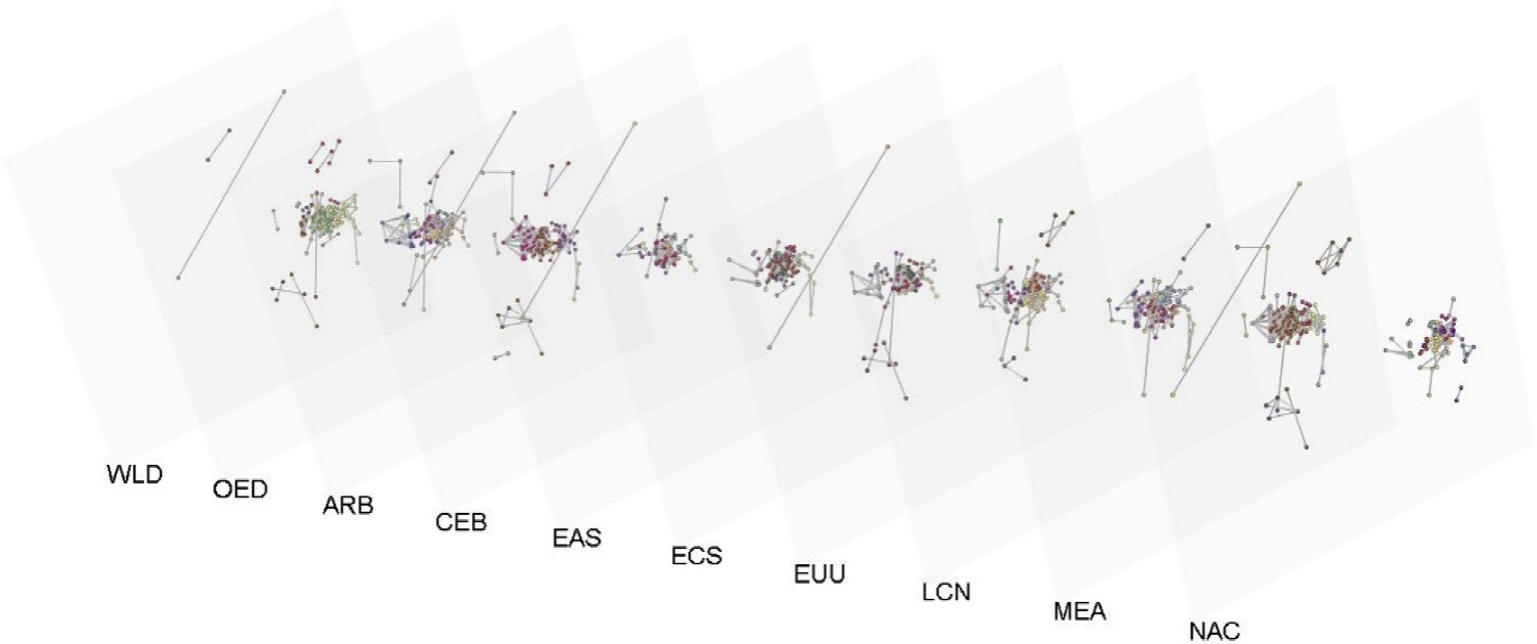
Illustration of how multiplex networks can highlight the differences between how the SDG indicators are interconnected in different countries and regions.

**Fig. 5:**
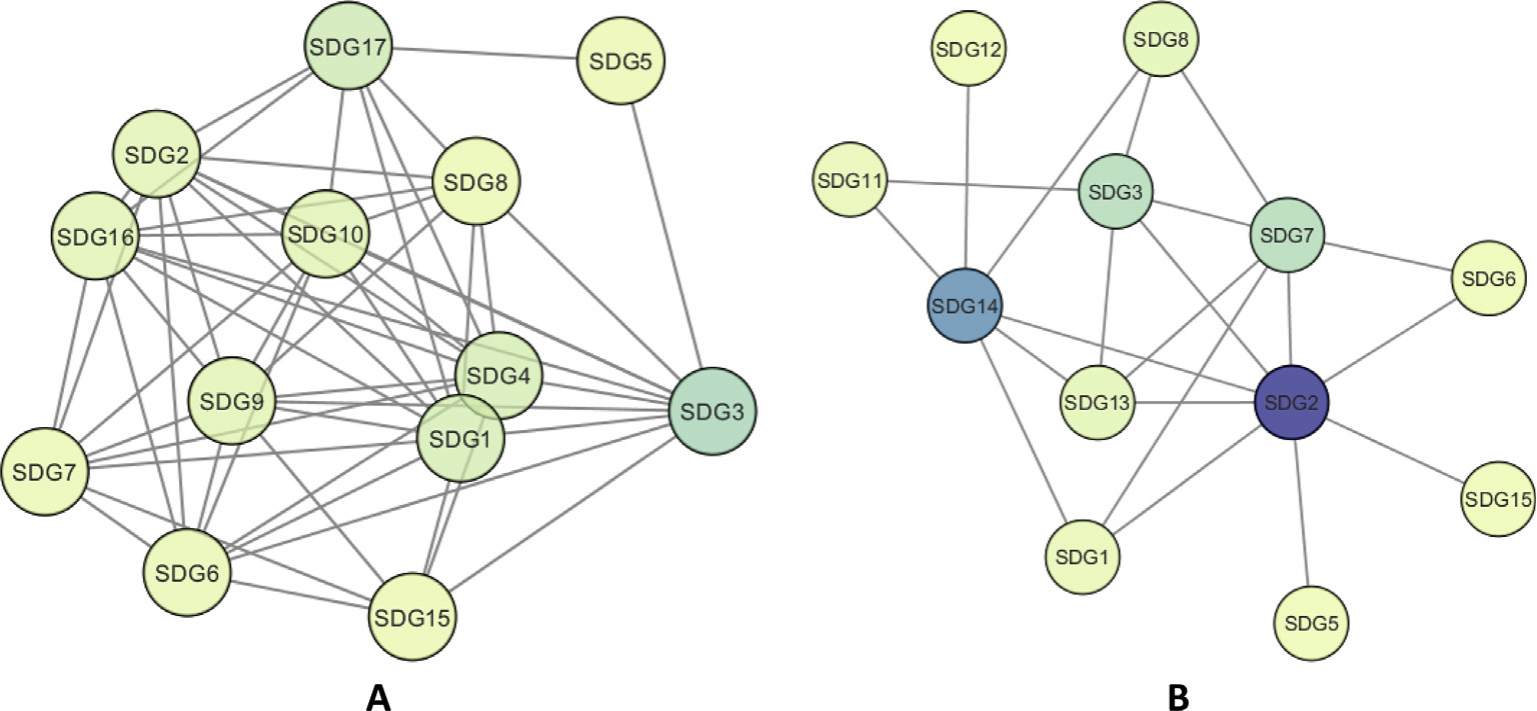
The differences between the networks of the linked SDG indicators (Part A) and the study of International Council for Science (Part B) shows how the proposed dataset enriches the knowledge about the interrelationships of the sustainability goals.

To illustrate national differences in sustainable development priorities, a multi-layered network of ten major regions of the world is shown on Fig. 4. The colors of the nodes represent the communities of the related indicators. Fig. 5 shows the relations among the sustainable development goals. The colors of the nodes represent the closeness centrality values. The A part of Fig. 5 is based on the study of the of International Council for Science and the B part is based on the correlation among the linked data.

## 2 Experimental design, materials, and methods

### 2.1 Linking the World Bank indicators with the SDG indicators

The most important step of the data generation was the assignment of the 1504 variables of the World Bank open data [1] to the 169 SDG indicators. This linking was performed by the authors based on their expertise. The targets (169) of the sustainable development goals (17) and the indicators are linked based on the experts of the Inter-Agency and Expert Group on Sustainable Development Goal Indicators [4], [5].

A part of the resulted network is illustrated in Fig. 1 that shows the linking of World Bank variables with the “Ensure availability and sustainable management of water and sanitation for all” development goal (SDG6). In Fig. 1 the blue colored circles show the SDG goals, targets and indicators. The shortened descriptions are inserted next to the circles. The grey colored circles are the World Bank Open Data variables. Linking variables and indicators has been achieved for all 17 goals. A variable has been linked to only one indicator, but an indicator can be described with multiple variables, so a total of 530 connections have been defined by the authors.

### 2.2 Exploring the internal linking the World Bank indicators

If we establish relationships among the variables of the World Bank (e.g.: based on the correlation), we indirectly link the sustainable development indicators, targets and goals.

Fig. 2 shows an example for indirectly connections of the SDGs. If the variable and SDG indicators have been connected the correlation among the variables also linking the targets and goals.

The interconnections of the SDG targets and goals help to identify the thematic areas of the sustainable strategic planning activities. In this data article a total of 908 correlation-based interconnections have been occurred.

The linking of the variables is based on the Pearson correlation coefficient values. (the variables were connected when the “ρ” exceeded 0.99 with *p* < 0.05 significance level. The availability of the variables varies regions by regions, so countries that have at least 20 years of data were taken into consideration. Considering the sign of correlations can help to explore synergies and compromises in the behaviour mapping of SDGs.

The correlation analysis and the generation of the input files of networks were performed with the published MATLAB/Octave program.

### 2.3 Multilayer network formulation of SDG indicators, targets and goals

The networks are also represented by their adjacency matrices. The linking of the World Bank dataset and SDG indicators are represented by the A_IV_ (indicator-variable; 241 × 530) adjanency matrix. The adjacency matrices of A_GT_ (goal-target; 17 × 169) and A_TI_ (target-indicator; 169–241) have been formulated also at Step 1. The correlation based relationships between the World Bank variables generated at Step 2. are stored in the A_VV_ (variable-variable; 530 × 530) matrix.

This representation is beneficial as it allows the modelling relationships among the targets and goals by multiplication of adjacency matrices. The following equations show the formulation of the target level interconnections A_TT_ (target-target; 169 × 169):

$$A_{TT}=\;{A^{T}}_{TI}\cdot {A^{T}}_{IV}\cdot A_{VV}\cdot A_{IV}\cdot A_{TI}$$

The interconnections of the SDG goals A_GG_ (goal-goal; 17 × 17) can be modelled with the projection of the A_GT_ (goal-target; 17 × 169) and A_TT_ (target-target; 169 × 169) adjacency matrices.

$$A_{GG}={A^{T}}_{GT}\cdot A_{TT}\cdot A_{GT}$$

The data article also generated a network based on the study of International Council for Science (ICSU) [6]. According to the ICSU study 238 positive, 66 negative and 12 neutral interactions were identified among the SDG targets, which are able for indirectly modelling of the SDG goals. The projection of the goal-level network is similar like in case of World Bank, nevertheless the relationships among the targets A_TT(ICSU)_ are already defined in the study.

### 2.4 Identification of key factors and thematic areas of SDGs

The key indicators of SDGs have been identified by centrality analysis of the networks and the clustering of the indicators has been done with community analysis in MuxViz environment. The networks of the SDGs have been attached to the dataset, therefore further investigations are possible.

The modelling steps are presented on Fig. 3. It should be pointed out, that the order of the steps shown in Fig. 3 are not interchangeable. Finally, a detailed analysis of the networks is done in MuxViz, where the country-level dissimilarities of the interconnections of SDGs can be examined, as well as communities and key indicators of SDGs can be identified.

A comparison of the inferred target-target networks and the network defined by experts from ICSU is depicted in Fig. 5. As can be seen the proposed data can provide a comprehensive view of how SDGs are interrelated, which interconnections are detailed in Ref. [2]. With the help of the proposed data and program not only this publication can be reproduced but further detailed analysis can be performed.
