# Supplementary material for: Data-driven multilayer complex networks of sustainable development goals
Source: Data Brief. 2019 May 23;25:104049. doi: 10.1016/j.dib.2019.104049 (PMC6554224; doi:10.1016/j.dib.2019.104049)
Supplement: Multimedia component 1 [file mmc1.pdf]

Conflict of Interest Declaration

We wish to confirm that there is no known conflict of interest associated with this publication and there has been no significant financial support for this work that could have influenced its outcome.

We confirm that the manuscript has been read and approved by all named authors and that there are no other persons who satisfied the criteria for authorship but are not listed. We further confirm that the order of authors listed in the manuscript has been approved by all of us.

We confirm that we have given due consideration to the protection of intellectual property associated with this work and that there are no impediments to publication, including the timing of publication, with respect to intellectual property. In so doing we confirm that we have followed the regulations of our institutions concerning intellectual property.

Behalf on the authorship,

Sincerely yours,

Viktor Sebestyén

Institute of Environmental Engineering

University of Pannonia (Hungary)
